# Letter to the Editor about “Burden trend and forecasting analysis of malignant skin melanoma from 1990 to 2021”

**DOI:** 10.1097/JS9.0000000000004646

**Published:** 2026-01-12

**Authors:** Huizi Li

**Affiliations:** Department of Oncology, The Affiliated Jiangning Hospital with Nanjing Medical University, Nanjing, Jiangsu, PR China

*Dear Editor,*

We read with great interest the recent study on the global burden trends and projections of malignant skin melanoma (MSM) from 1990 to 2021^[1]^. The authors utilized data from the Global Burden of Disease (GBD) 2021 dataset to comprehensively assess 204 countries across various regions and sociodemographic index (SDI) levels. By integrating prevalence, incidence, and disability-adjusted life years (DALYs), and applying joinpoint regression together with ARIMA modeling, the study provides valuable insights into the temporal patterns and future trajectories of MSM worldwide. Nevertheless, while the research offers important epidemiological information, several key methodological and interpretative issues merit further consideration.

First, the limitations of data sources appear to be underestimated. Although the GBD database is comprehensive, it relies heavily on Bayesian modeling and multiple heterogeneous data inputs such as national surveys, hospital records, and literature reviews. In low-SDI regions (e.g., Southeast Asia and sub-Saharan Africa), these estimates are particularly prone to underreporting and misclassification. The authors acknowledge that the low disease burden in such regions may partly reflect “underdiagnosis due to limited healthcare infrastructure,” yet the magnitude of this bias remains unquantified. For example, the age-standardized prevalence in Oceania was reported as only 0.3 per 100 000 is this a true reflection of low incidence, or a systematic artifact of data collection? Similarly, the markedly higher rates observed in high-SDI regions such as Australasia (age-standardized incidence of 32.4 per 100 000) may overstate the actual burden. Explicitly estimating the impact of underreporting, or performing sensitivity analyses to assess the robustness of results, would substantially strengthen the study’s conclusions.

Second, although the paper discusses ultraviolet exposure, sex, and genetic susceptibility as potential risk factors, these associations remain descriptive rather than empirically tested. For instance, the reported increases in MSM burden in Eastern Europe and East Asia are attributed to “lifestyle changes” and “urbanization,” yet no data are provided to substantiate these claims. Incorporating socioeconomic, environmental, and behavioral covariates into the analysis would enhance the interpretability and explanatory power of the findings.

Finally, while the authors highlight the need for sex- and SDI-specific intervention strategies, they stop short of proposing actionable measures. Given the higher burden among men and in high-SDI regions, the study could have suggested tailored screening programs, public health campaigns, or frameworks for resource allocation. The absence of such discussion limits the practical value of the findings for policy development. Furthermore, the observed decline in DALY rates is attributed to advances in immunotherapy and targeted therapy; however, this causal link is not empirically demonstrated^[2]^. For example, the total number of DALYs increased from 1.046 million in 1990 to 1.679 million in 2021, reflecting population growth and aging rather than purely therapeutic improvement.

In summary, this study makes a meaningful contribution by elucidating the global dynamics of MSM burden. Nevertheless, greater methodological rigor and deeper contextual interpretation are needed to mitigate potential biases and ensure relevance for public health decision-making. We hope our comments will be useful for future revisions and strengthen the article’s impact in guiding global efforts to reduce the burden of MSM. This correspondence was prepared in accordance with the TITAN guidelines^[3]^.

## Data Availability

Not applicable.

## Research registration unique identifying number (UIN)

There are no conflicts of interest.
